# A framework model for current land condition in Iceland

**DOI:** 10.1371/journal.pone.0287764

**Published:** 2023-07-06

**Authors:** Ólafur Arnalds, Bryndís Marteinsdóttir, Sigmundur Helgi Brink, Jóhann Þórsson

**Affiliations:** 1 Agricultural University of Iceland, Reykjavík, Iceland; 2 The Soil Conservation Service of Iceland, Reykjavík, Iceland; Universidade de Lisboa Instituto Superior Tecnico, PORTUGAL

## Abstract

Iceland border the Arctic with cold maritime climate and a large proportion of the land placed at highland plateaus. About 1100 years of human disturbance, such as grazing and wood harvesting, has left much of the island’s ecosystems in a poor state, ranging from barren deserts to areas with altered vegetative composition and degraded soils. We constructed a novel resilience-based model (RBC-model) for current land condition in Iceland to test which and how factors, including elevation, slope characteristics, drainage, and proximity to volcanic activity, influence the resilience and stability of ecosystems to human disturbances. We tested the model by randomly placing 500 sample areas (250 x 250 m) all over the country and obtaining values for each factor and current land conditions for each area from existing databases and satellite images. Elevation and drainage explained the largest portions of variability in land condition in Iceland, while both proximity to volcanic activity and the presence of scree slopes also yielded significant relationships. Overall, the model explained about 65% of the variability. The model was improved (R^2^ from 0.65 to 0.68) when the country was divided into four broadly defined regions. Land condition at the colder northern peninsulas was poorer at lower elevations compared to inland positions. This novel RBC model was successful in explaining differences in present land condition in Iceland. The results have implication for current land use management, especially grazing, suggesting that management should consider elevation, drainage, slopes and location within the country in addition to current land condition.

## Introduction

Land degradation is among the most severe environmental challenges facing mankind [1], and is at the center of all three major UN environmental conventions: Convention on Climate Change UNFCCC, Convention on Biodiversity (UNCBD), and the Convention to Combat Desertification (UNCCD). Land degradation has had a severe effect on biodiversity and has contributed a large proportion of the greenhouse gases that threatens Earth’s climate [2]. Grazing by livestock is a major contributor to terrestrial degradation processes and perhaps more so than any other single human activity [1].

**Fig 1:**
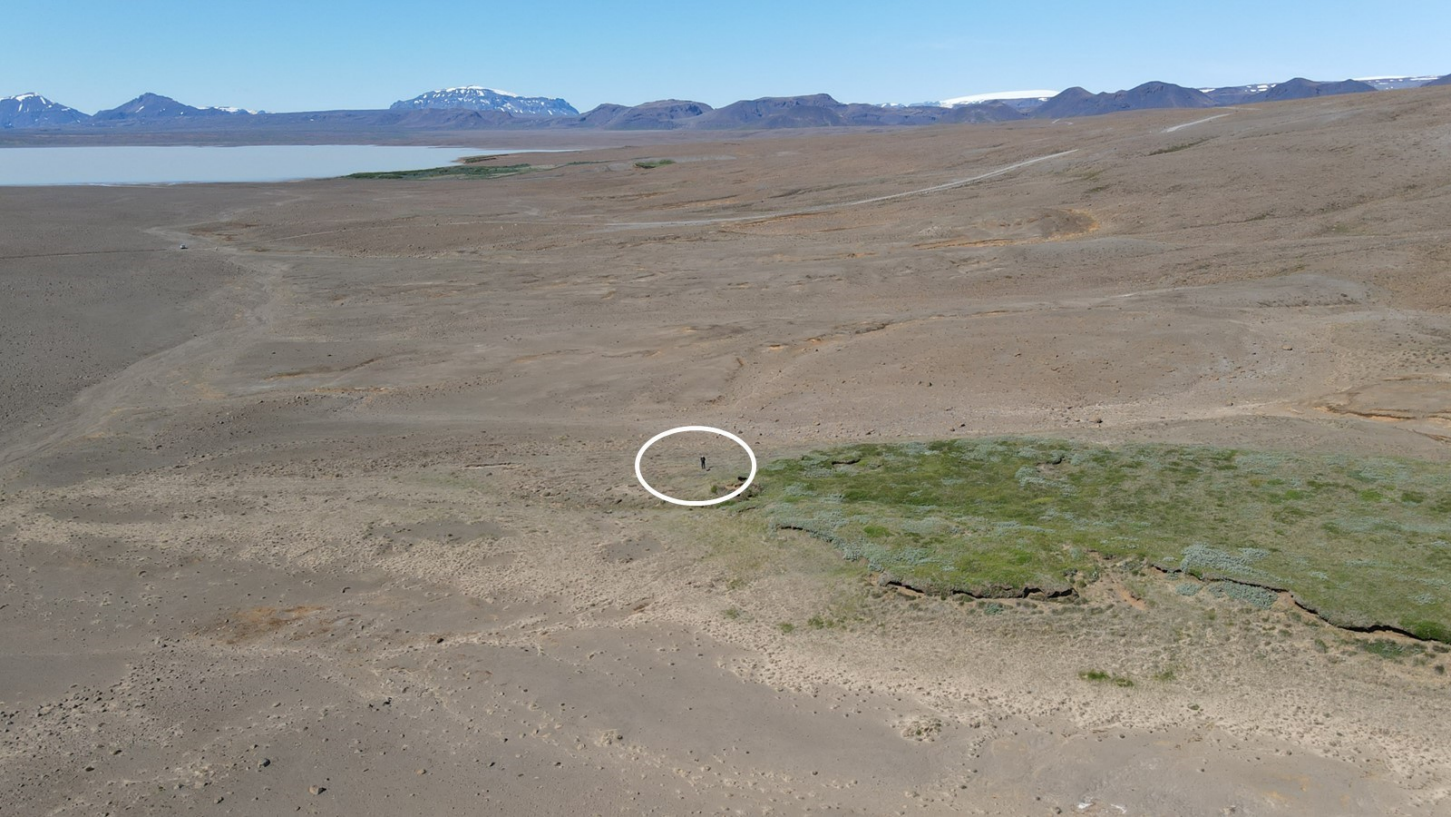
Severely degraded landscape in South Iceland–a ‘desert’. Remnants of deep soils (Andosols) and vegetation as an island on the right in the barren landscape. Glacial till surface from the Pleistocene glaciation left behind after soil erosion has removed the fertile dark-colored Andosols. The surface has been lowered by ~2.5 meters. The person to the left, inside the white circle, of the vegetation island provides scale. The area was previously fully vegetated with birch forests, which were utilized as fuel into the 17^th^ century. The soils were coarse grained, near the active volcanic belt and susceptible to erosion.

Iceland, a volcanic island in the North-Atlantic Ocean, was settled by Norsemen and Celts in the latter part of the 8^th^ Century [3]. Since then, Icelandic ecosystems have undergone severe land degradation resulting in systems in poor land condition [4–8]. Iceland has sometimes been rated among Earth’s most severely affected areas of land degradation [9, 10]. Recent survey of land condition in Iceland has reaffirmed the poor land condition [11]. Degradation processes include wind and water erosion removing entire soil mantles from the landscapes, advancement of sand burying or destroying existing systems (“sandification”), woodland destruction by grazing and wood harvesting, and degradation of soil and vegetation systems including transition to lower ecological states, mostly through grazing by domestic animals [5, 12, 13]. The most severe forms of degradation have resulted in the formation of extensive barren surfaces, even in areas previously covered by birch woodlands (Fig 1).

Ecosystem degradation in Iceland has been subjected to multiple research efforts employing a variety of methods [reviewed by 7]. Diverse sources indicate past ecosystem riches (see S1 Table). They are described in old written documents like sagas and annals [7, 14, 15]), vegetation and soil remnants indicate richer ecosystems [7] as well as rich sites protected from grazing [7, 16], multiple evidence of past riches and farms in presently severely degraded areas both from places name [14] and archeological evidence [17, 18], and modelling former birch distribution which indicates a wide spread of birch forests and shrubs before the settlement [19]. There is also evidence of land degradation following the settlement. Pollen research indicate dramatic vegetation changes at the time of settlement [18, 20–22] and aeolian deposition rates increased 4–10 times after the settlement than before due to wind erosion of soils and gradually larger desert areas [23]. The degradation probably occurred at different time scales after the settlement in the late 9^th^ century, as indicated by sedimentation rates documented in soils and lake sediments [24–26]. The current state of Icelandic ecosystems is also variable throughout the country [11; see also Fig 3], indicating variability in degradation both in time and space. The link between ecosystem resilience, natural stresses such as volcanic ash deposition and cold spells on one hand and continued land use on the other has been cited by many in relation to explaining the degradation of Icelandic ecosystems [13, 27]. Land use has been quoted as the most important factor, reducing, or even dwarfing the effects of the cooling during the Middle Ages [28–30]. However, land use effects over 1100 yrs of settlement vary within and between regions [7, 26], and some debates have been associated with the relative importance of climate effects compared to other factors influencing land condition [31].

Both the variable current condition and different periods of degradation have sometimes led to contradicting statements about the causes of the degradation–including debates about the role of land use in the processes [8]. It is important for interpreting current land condition to relate natural factors with ecosystem responses to long-term land use pressures. Here we present a novel resilience-based model to explain the current condition of Icelandic ecosystems. It is based on several factors that are likely to influence the resilience and stability of ecosystems in relation to land use, which are considered to be mainly grazing and woodland destruction. The factors of the model are **elevation above sea level, proximity to the active volcanic zones, drainage (presence of wetland) and slope characteristics.** The model serves as an example how studying independent natural factors and employing available databases can improve understanding of current land condition in degraded areas. The model is important to identify means to reduce negative impacts of land degradation, adjust land use pressures and reverse degradation trends.

## Materials and methods

### Study site

Iceland borders the Arctic Circle with subarctic and arctic climate conditions in highland areas while the climate of the lowlands below 200 m elevation can be described as cold, maritime boreal. The Gulf Stream brings relatively warm waters to Iceland [32] resulting in mild winters (-5°C on average) and cool summers (7°C on average) [33]. Annual precipitation ranges broadly from 400 mm in North Iceland to > 2,000 mm on mountain slopes in South Iceland [34]. Strong winds characterize the weather and frequent weather fluctuations, with numerous passages of low-pressure areas near or over the island [35].

Most inhabitants live in the lowlands below 200 m elevation which make up about 24% of Iceland, and this is where most of the agriculture areas are located [36]. Areas 200–400 m above sea level (18%) can be productive inland but can be considered marginal areas at more oceanic locations, hence the cool summers and northerly location, which is reflected in studies of vegetation indices on satellite images [37]. All areas above 400 m.a.s.l. (58%) can be considered highlands, with a large proportion (37%) of the country being above 600 m elevation. Volcanic activity provides the parent materials of the soils in Iceland together with aeolian sediments (dust) from various sources which continuously are added to the top of the soils. The soils where vegetation cover remains are often 1–2 m thick, coarse-grained Andosols in dryland positions, with limited cohesion and quite susceptible to erosion by wind and water [7]. Icelandic wetlands are a rare mixture of Andosols and peat soils (Gleyic and Histic Andosols, and Histosols) [38].

The soils of the barren surfaces–often referred to as sandy ‘deserts’ are classified as Vitrisols, separating them from classical Andosols, according to the Icelandic classification scheme [7]. The Vitrisols lack organic content and water holding capacity and dry out easily due to their dark basaltic surfaces. Barren surfaces cover about 40 000 km^2^ or 40% of Iceland’s land mass. Some of the sandy areas, such as floodplains at the margin of glaciers and shorelines near outlets of glacial rivers serve as major sources of sand and dust [39]. Advancement of sand is responsible for the destruction of extensive ecosystems throughout historical times in Iceland.

Vegetation of Iceland is characterized largely by heathlands and ‘mossland’ in various condition (about 35% of the land) and a range of barren areas (about 40%) while shrubs and forests are only about 1.5–2% and agricultural land <2% (mainly hayfields). Grassland is considered about 2.3% of the land cover. Wetlands make up 6–10% of the country, the value dependent on databases used [7].

### A resilience-based model for land condition; RBC-model

Here we introduce a simplified resilience-based framework model for land condition (RBC-model) using readily available data for the tested factors. A preliminary and more complex outline of the model was presented in Icelandic by Arnalds [8].

**Fig 2:**
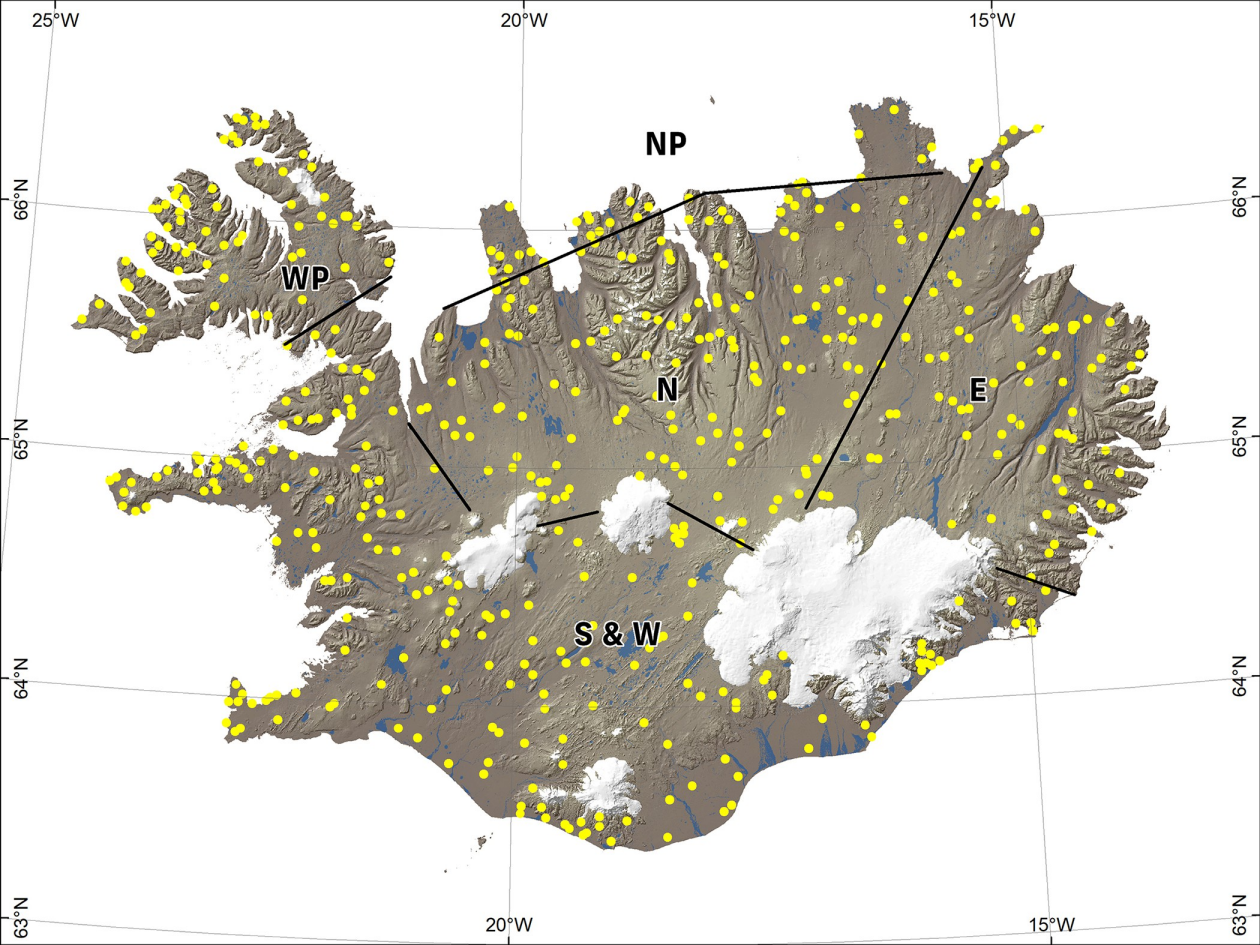
Distribution of the 500 sample areas (SA’s) in Iceland. The lines separate geographical areas used in some of the statistical analysis. The Southern and Western region (S&W), the Northern region (N), Eastern region (E) and the Peninsulas (P). The peninsulas consist of the northernmost part of Iceland (NP) and the Westfjord Peninsula (WP).

In the model, elevation above sea level, proximity to the active volcanic zones, drainage and slope characteristics are used to explain current land condition. We also tested if the relationship was different based on regions of the country. To obtain data for the model, each of the above factors and land conditions were accessed in 500 sample areas (SA) around the country. In order to position each SA, a 500 x 500 m grid system was placed over all Iceland using ArcGIS Pro software [40]. Subsequently squares with water bodies (rivers and lakes) and glaciers were excluded, leaving 318,803 squares on the surface. Out of these, 500 were randomly selected as the sample areas (SA‘s) in the study (Fig 2), each being 500 x 500 m square, or 0.25 km^2^ (250 ha).

Each of these 500 SA’s was paired with various geographical databases for determining components of the model presented in Table 1. When a SA was partially covered with snow or water, that proportion was excluded from calculations for each plot. Furthermore, each of the SA’s were examined visually on SPOT satellite images which reduced the total number of SA’s used for statistical analysis by 28 points, down to 472 used in the dataset (total of 118 km^2^, 0.12% of Iceland). Criteria for exclusion included built up environments and areas subjected to continuous disturbances such as by river flooding, sandy ocean shorelines and newly exposed areas after glacial retreat due to global warming. In these areas land condition will be highly affected by these disturbances but not by the factors tested in this study. One SA was omitted because of unexplained error in the database that was used to assess land condition.

**Table 1:** List of variables extracted for each of the 500 sample areas (SA’s) in Iceland. Further explanation is provided for each variable in the text.

Variable	Notes
**GL-value (**GroLind average value for each SA)	Geographic database for ecosystem functioning and stability. A proxy for present land condition, the response value for the model.
**Elevation**	Average elevation of 5x5 m raster pixels within the 500 x 500 m SA’s
**Dust sedimentation rates**	A proxy for distance from the active volcanic zone and active sources of sand.
**Drainage** (wetland vs dryland)	Based on Nytjaland* GIS database and direct observations on SA´s on satellite images.
**Slope** (angle, %)	Average slope angle within each SA
**Scree slopes**	Determined by zooming in on satellite images for each of the SA’s
**Region**	A coarse division of Iceland into four geographical regions

* [48]

In this study we did not assess temporal, regional or within region differences in land use intensity over the past 1100 years. Even though land use is an important factor, influencing current land condition, limited records are available about current and past land use for the whole of Iceland. Type of land use and pressures are bound to have changed from the time of settlement to the present, subsequent to ecosystem changes that in part were caused by the use of the land. We assume that pressures have been high in most regions at some point in time as the survival of humans expressed by population numbers were highly dependent on the number of grazing animals for food production [3]. It should be noted that at higher elevations, with lower productivity and vegetation yields, relatively low animal density would have resulted in intense land use pressures in areas of low resilience because of climatic constraints. Various other factors have affected the land use pressures throughout the ages, including the possibility of making hey for winter fodder, which supports more livestock through winter–hence higher grazing pressures in summer.

### The variables used in the model

Variables identified for each of the SA’s are listed in Table 1 and discussed in further detail below. They are based on independent sources which include direct observations by zooming in on satellite images on each SA.

#### Land condition: GroLind database and GL-values

**Fig 3:**
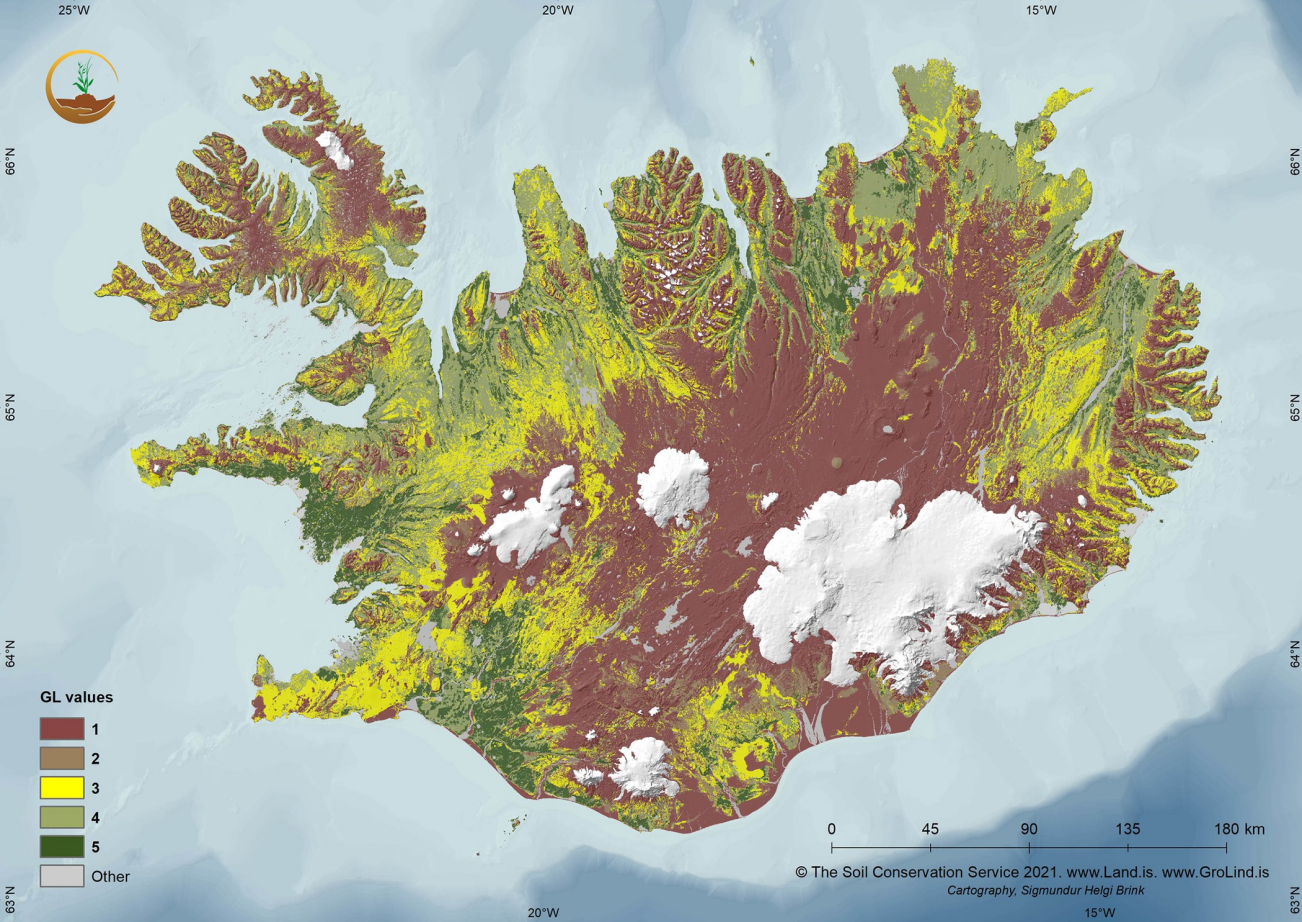
GroLind land condition (GL-value) map for Iceland. The codes for the values are 1: very poor; 2: poor; 3: marginal; 4: good; 5: excellent (see also Table 2). © The Icelandic Soil Conservation Service/GroLind.is.

Land condition, the response value of the RBC-model, was represented by a recently published land condition survey titled ‘GroLind’ [11]. Land condition was obtained by extracting information from a recent mapping of habitat types of Iceland (pixel size 5 x 5 m), based on the EUNIS classification [41] and a coarse scale older mapping of soil erosion in Iceland (1:100 000 scale) [42]. From these maps five factors were extracted that reflect ecosystem conditions. Four were from the habitat type map, average i) vegetation height, ii) proportion of bare ground, iii) proportion of vascular plants and iv) carbon content in soil surface horizons and v) soil erosion severity, from the soil erosion map. Each of these factors were assigned ‘ecosystem function grade from 1 (e.g. low vegetation or low cover of vascular plants) to 5 (e.g. high vegetation). Erosion was rated on the scale 1–10, thereby giving areas with limited erosion more emphasis in comparison to the other four factors rated on the 1 to 5 scale. By summarizing grades of all five factors for each raster pixel, a cumulative grade was obtained for land condition, ranging from 5 (all factors rated in poor condition) to 30 (all factors rated in excellent condition) (Table 2). The cumulative grade was then divided up to five land condition values (GroLind value or “GL-value”) from 1 (Very poor conditions) to 5 (Excellent conditions). An overall GL-value map for Iceland is presented in Fig 3. Each pixel of the map thus had a GL-value (condition value) ranging from 1 to 5.

**Table 2:** GL-values based on a land condition survey in Iceland. The values are based on cumulative grade range for five factors: i) vegetation height, ii) proportion of bare ground, iii) proportion of vascular plants and iv), carbon content in soil surface horizons and v) soil erosion. Each grade range results in a GL-value, ranging from 1 (very poor condition) to 5 (excellent condition).

GL- value	1	2	3	4	5
**Land condition**	Very poor	Poor	Marginal	Good	Excellent
**Function and stability**	Limited ecosystem function and stability		Marginal	Robust ecosystem functions and stability	
**Cumulative grade** ^**$**^	5–12	13–16	17–21	22–26	27–30

$: Total by addition of the grades for vegetation height, bare ground, vascular plant cover, soil carbon content and erosion

**Fig 4:**
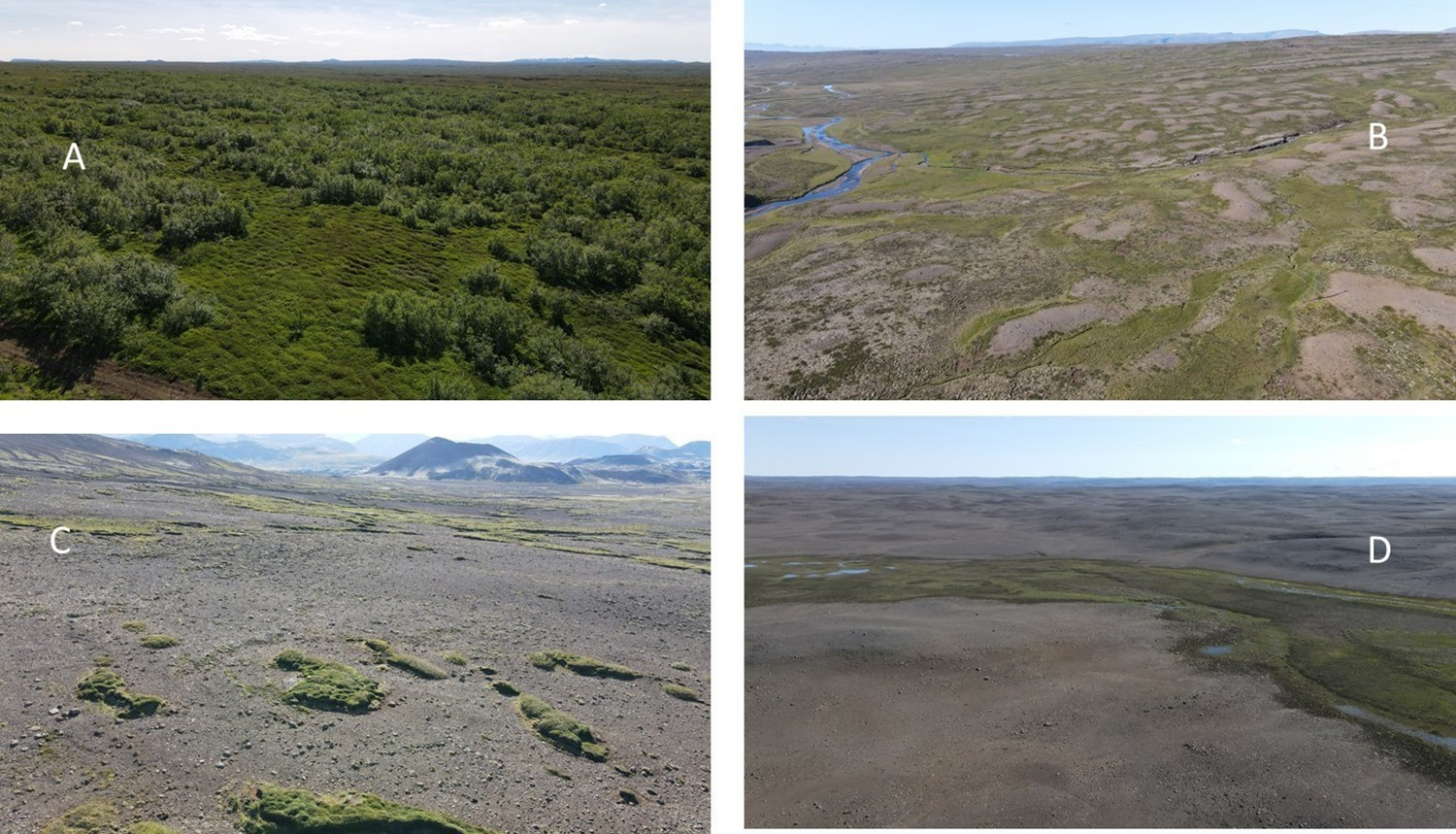
Examples of land condition in Iceland. A: Excellent, native birch forests (GL-value 5. B: Degraded, about 60% vegetation cover, marginal to poor land condition (GL-values 2 and 3). C: Very degraded area, vegetation islands remain, very poor land condition (GL-value 1). D: Desert area with vegetation remains in wetter depression. Mixture of good condition (wetland–GL-value 4) and very poor condition (desert–GL-value 1). Depending on how the sample area (SA) is selected, the land in figure D can have an average GL-value of 2–3.

Each SA typically consisted of entities (pixels) of more than one GL-value. A mean GL-value, was calculated for each of the 500 x 500 m sample areas (SA’s) which represents the land condition response value of our model. The values range from 1 (poor land condition, functioning and stability) to 5, which excellent land condition with good ecosystem functioning and stability (see examples in Fig 4). It clearly indicates that the highland interior areas often have the lowest grade, which in part consists of sandy barren surfaces (‘deserts’). Same applies for the Westfjord Peninsula of Northwest Iceland. Higher GL-values are encountered in the flat lowlands of the South and West, which chiefly consist of wetland areas. According to the GroLind classification, about 45% of the land area is classified as having limited ecosystem function and stability, 15% marginal land and 26% with robust ecosystem functions and stability, the remaining 14% are glaciers, water and man-made surfaces. The GL- values of our SA’s, represented well this distribution of land condition of the country.

#### Elevation

Average summer temperatures become lower with elevation [33, 35], thus limiting photosynthesis, plant growth and the resilience of ecosystems at higher elevations. Therefore, elevation is likely to influence the resilience and stability of ecosystems in relation to land use. Elevation was determined as the mean elevation for each of the SA‘s using the ArcticDEM elevation data [43] employing the ArcGIS Pro software.

#### Proximity to the active volcanic zones

Volcanic eruptions both apply direct negative impacts on ecosystems and influence the susceptibility of the soil systems with thick coarse soils more susceptible to massive erosion [7]. The impact of ash-fall events is influenced by the nature and resilience of the system receiving the impact, determined by such factors as amount of ash, the height of the vegetation, and the overall state of the ecosystem [44–46].

**Fig 5:**
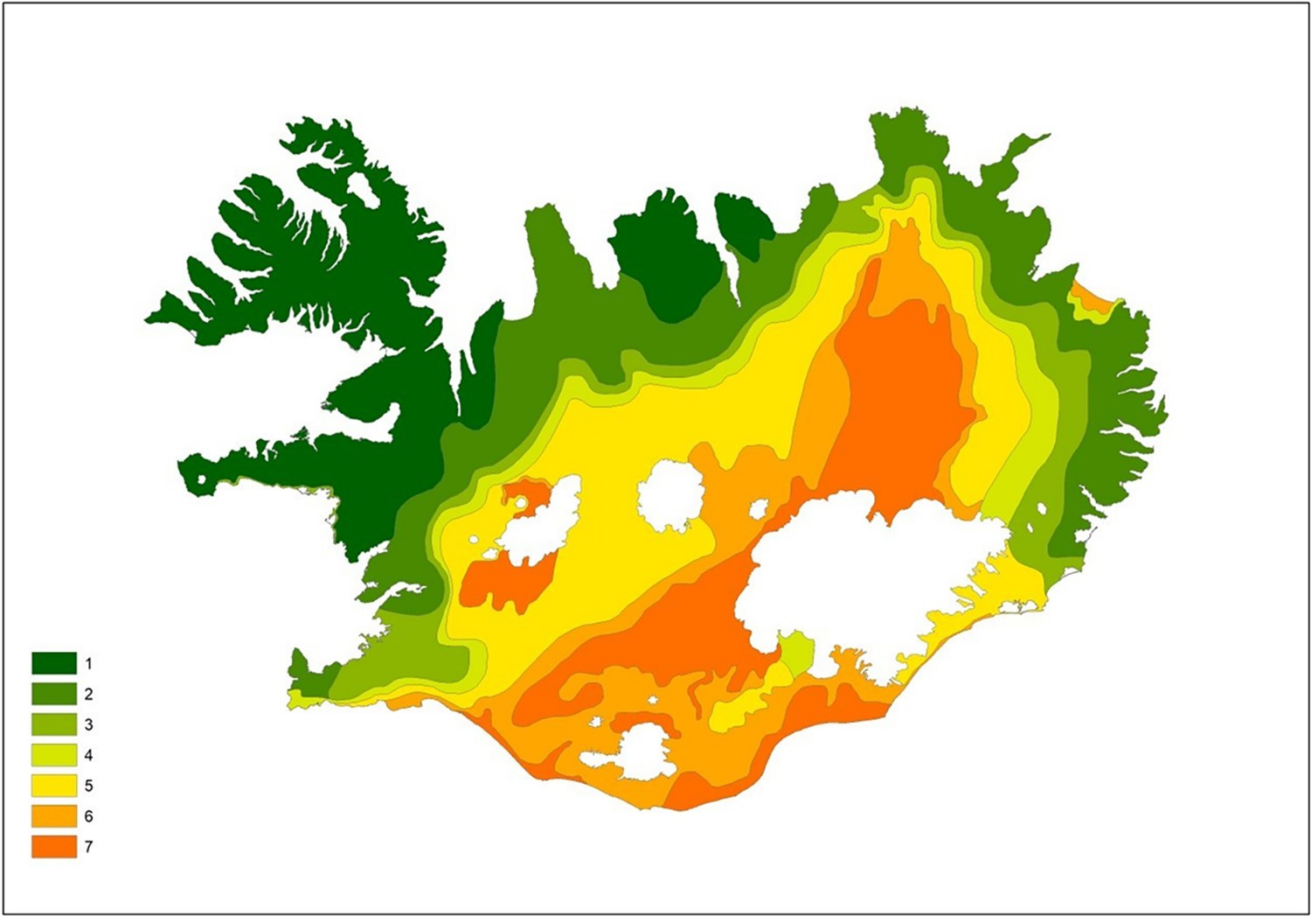
Map of dust deposition rates in Iceland from very low (1) to excessive deposition (7). 1: <50; 2: 50–100; 3: 100–150; 4: 150–250; 5: 250–500; 6: 500–1000; 7 >1000 g m^-2^ yr^-1^. Based on [39, 47].

The rates of dust deposition served as a representation for the proximity to the active volcanic belt–with growing probability of ash fall events, and the location in relation to sandy deserts–which today are the major sources of the dust [39]. Dust sedimentation rates were obtained from a GIS database for dust deposition [39, 47] (Fig 5). Dust deposition rates were separated into seven classes, with rates shown on the legend of Fig 5. During peak times of ecosystem destruction in earlier times, dust also originated from redistribution of soils by wind erosion associated with collapse of vegetated systems, which subsequently became barren areas [see 7]. The thick coarse-grained soils that are most vulnerable to wind erosion were mostly associated with near or within the volcanic belt, which reinforces testing of this variable for our resilience-based land condition model.

#### Drainage/Wetlands

Wetlands are continuously moist and are therefore usually not prone to wind erosion. Flat landscape positions reduce susceptibility to water erosion and the wetness supports relatively robust vegetation cover at most times. Thus, the wetlands are relatively resilient systems compared to other land ecosystems. A wetland index was determined as the proportion of wetlands within each sample area (SA’s). The proportion was based on the distribution of the wetland and semi-wetland distribution of the Nytjaland land cover classification [48] and further verification by examining satellite images for each plot. The Nytjaland classification is based on classification of satellite images into eight relatively broadly defined vegetation classes. The wetland and semi-wetland categories both represent aquic soils. About 70% of the lowland wetlands have been drained for haymaking for winter fodder [38] and drained wetlands often classify as grasslands in databases. This was accounted for by zooming in on the satellite images of each plot and noting the presence of drainage ditches, surface water and other signs of a high water table.

#### Average slope angle

Ecosystems on steep slopes are less resilient to disturbances due to their instability and susceptibility to water erosion. Long continuous slopes are normally more prone to high erosion rates than shorter ones, which is reflected in water erosion models [49]. The mean slope (degrees) within each of SA was obtained by a planar method for calculating the rate of change in value from a cell to its immediate neighbor. The calculation was preformed on a projected flat plane using a 2D Cartesian coordinate system employing the slope tool in the ArcGIS Pro [50].

#### Scree slopes

**Fig 6:**
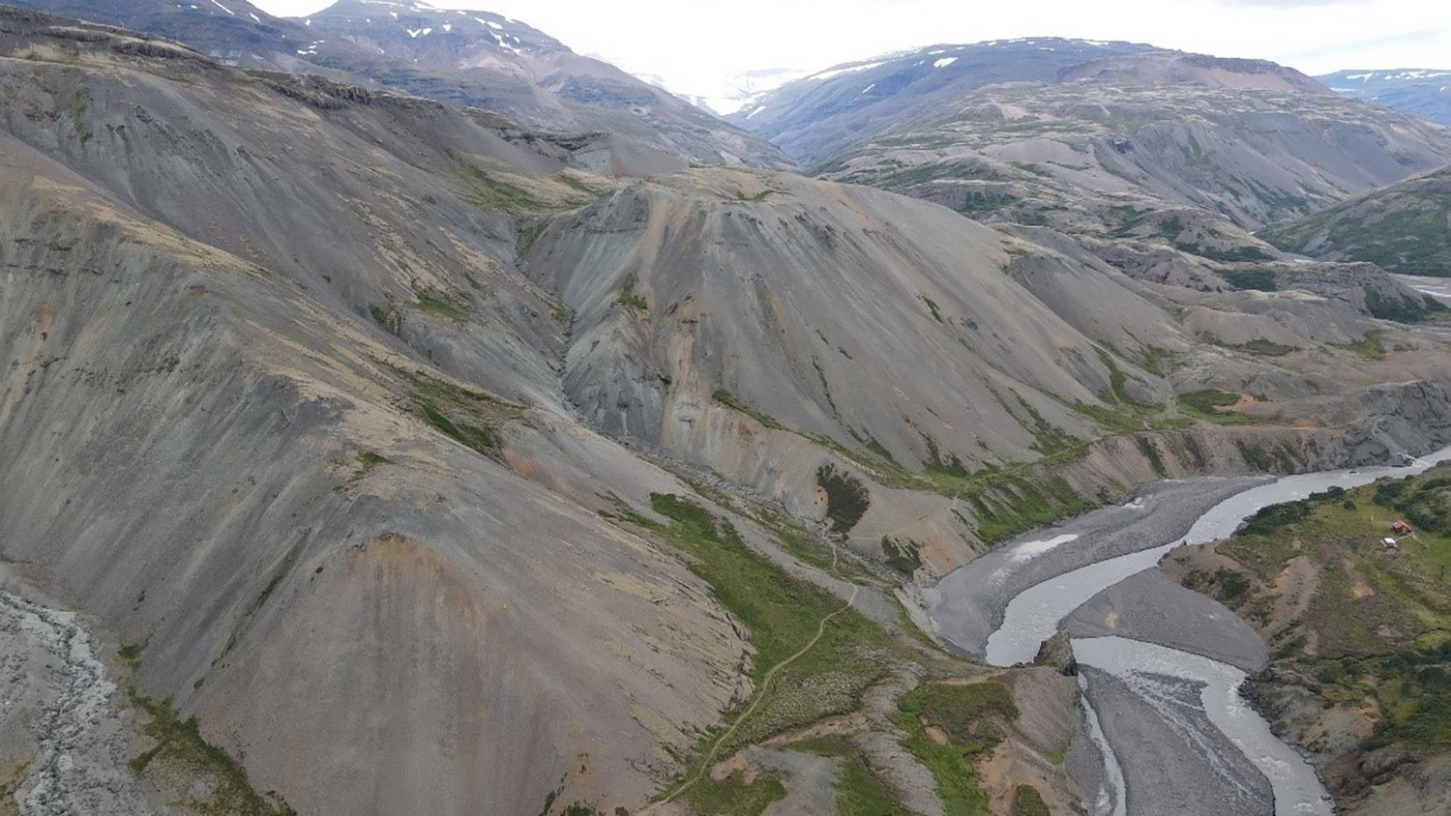
Scree slopes in SE-Iceland. Such landscapes have long continuous and often steep slopes. Composition is commonly rhyolitic associated with extinct volcanic systems, in contrast to the basaltic lava flows making up most Icelandic rocks.

Scree slopes, which are steep barren slopes covered with relatively loose coarse gravel, often 1–100 cm in diameter (see Fig 6). When the slopes had vegetation cover and soil mantle prior to the settlement, they would have been long continuous slopes, which increases the probability of severe water erosion. These slopes would therefore have been quite susceptible to land use pressures and especially the removal of the woody plant-cover by harvesting and grazing. Bare scree slopes are quite common in many areas of steep terrain, often associated with centers of extinct volcanic systems (‘central volcanoes’) in the Tertiary rock formations [51]. The extinct central volcanoes tend to be more rhyolitic (higher SiO_2_) and gravelly compared to the basaltic composition of the Tertiary lava stack–which often has shorter slope segments broken up by the individual lava formations, and a higher moisture content. Scree slopes were determined by visual identification on satellite images of each SA, given average rating from 1–5 based on the erosion mapping grading system for Iceland [42].

#### Regions

Different regions of Iceland have different climatic conditions and therefore are likely to vary in resilience [33]. There is no official separation of Iceland into geographical regions. We made a simple division into regions (Fig 2) partly based on traditional geopolitical separation of Iceland. The Southern and Western region, the Northern region, Eastern region and the Peninsulas. The peninsulas consist of the northernmost part of Iceland (NP) and the Westfjord Peninsula (WP in Fig 2). These were joined into a separate category, as climate there is expected to be colder due to proximity to the Arctic Ocean [33] and therefore may give a different response for elevation.

### Statistical analysis

All statistical analysis were made in R-GUI [52] using the additional packages lm.beta [53], ggplot2 [54] and Jtools [55].

Multiple regression analysis was used to test if elevation, dust sedimentation rates, drainage/wetland, scree slope, slope angle and region was significantly related to GL-values reflecting current land conditions. Model selection was done using backward selection and the Akaike Information Criterion (AIC) used to identify the best model [56]. Both normal coefficients (β) and standardized coefficients (*β-standard*) were calculated for each variable in the model. To visualize the relationship between each significant predictor variable and land condition we did a partial residual plots, which plots the predictor against the observed data with the effects of all the control variables accounted for [57]. Each region of Iceland was also tested separately with a multiple regression analysis to determine if model predictions differed between regions.

## Results

**Fig 7:**
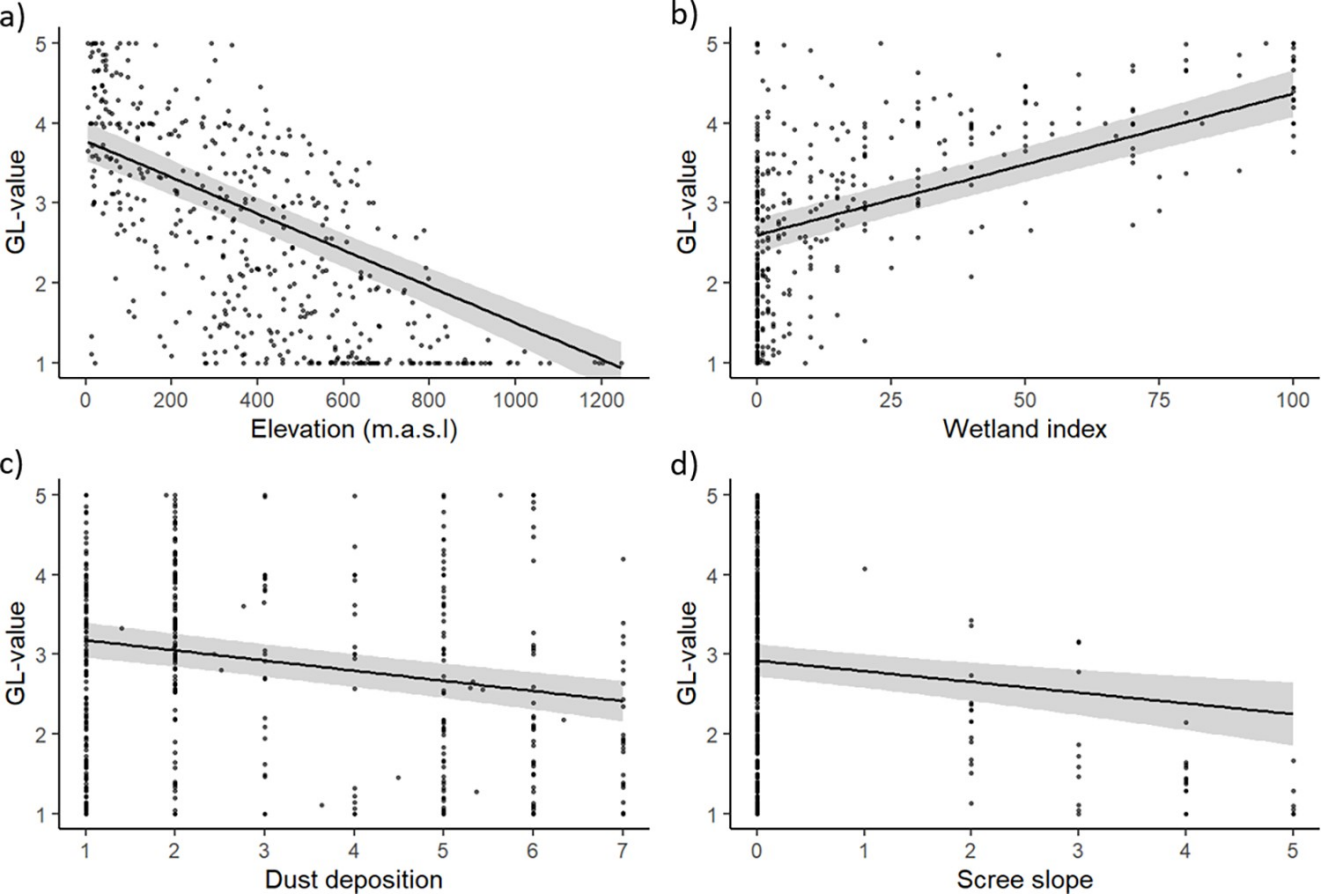
A partial residual plot on the relationship between land condition values (GL-value) and A) Elevation (m.a.s.l.), B) Wetland index/Drainage, C) Dust deposit and D) Scree slop in Iceland, based on 500, 500 x 500 m sample areas. The black line is the regression line and and the gray area represent 95% confidence interval around the regression line.

Majority of the observed variability in land condition in Iceland could be explained by the modelled factors and the best model included elevation, occurrence of wetlands, dust deposit, scree slope and region as the explanatory factors (F _(7.463)_ = 141.3, adjusted R^2^ = 0.676, p<0.001). Elevation and occurrence of wetlands explained the largest part of the variance (standardized β = -0.49, β = -0.002, p<0.001 and standardized β = 0.38, β = 0.02, p< 0.001, respectively). Dust deposition (standardized β = - 0.22, β = -0.13, p< 0.001) and scree slopes (standardized β = -0.11, β = -0.13, p< 0.001) also explained a significant part of the variability (Table 3, Fig 7). Including regions as a dummy variable in the model increased the predictability of the model (R^2^ = 0.646 without to R^2^ = 0.676 with regions).

**Table 3:** Results from a regression model explaining the relationship between land condition and elevation, dust sedimentation rates, drainage/wetland, scree slope and slope angle for each region in Iceland. Only results from the best model are shown. β st. is the standardized β.

	Adjusted R^2^	Elevation		Dust		Wetland		Scree	
		β st.	β	β st.	β	β st.	β	β st.	β
**South &West**	0.699	-0.45**	-0.002	-0.24**	-0.13	0.45**	0.018	-0.08 (*)	-0.13
**North**	0.687	-0,44**	-0.002	-0,32**	-0.21	0.22**	0.010	-0,16*	-0.27
**East**	0.658	-0,49**	-0.002	-0,20*	-0.17	0.49**	0.024	NS	
**Peninsulas**	0.636	-0.43**	-0.002	0.15*	0.35	0.46**	0.022	NS	

**: p < 0.001

*: p < 0.05 (*): p = 0.05–0.1

**Fig 8:**
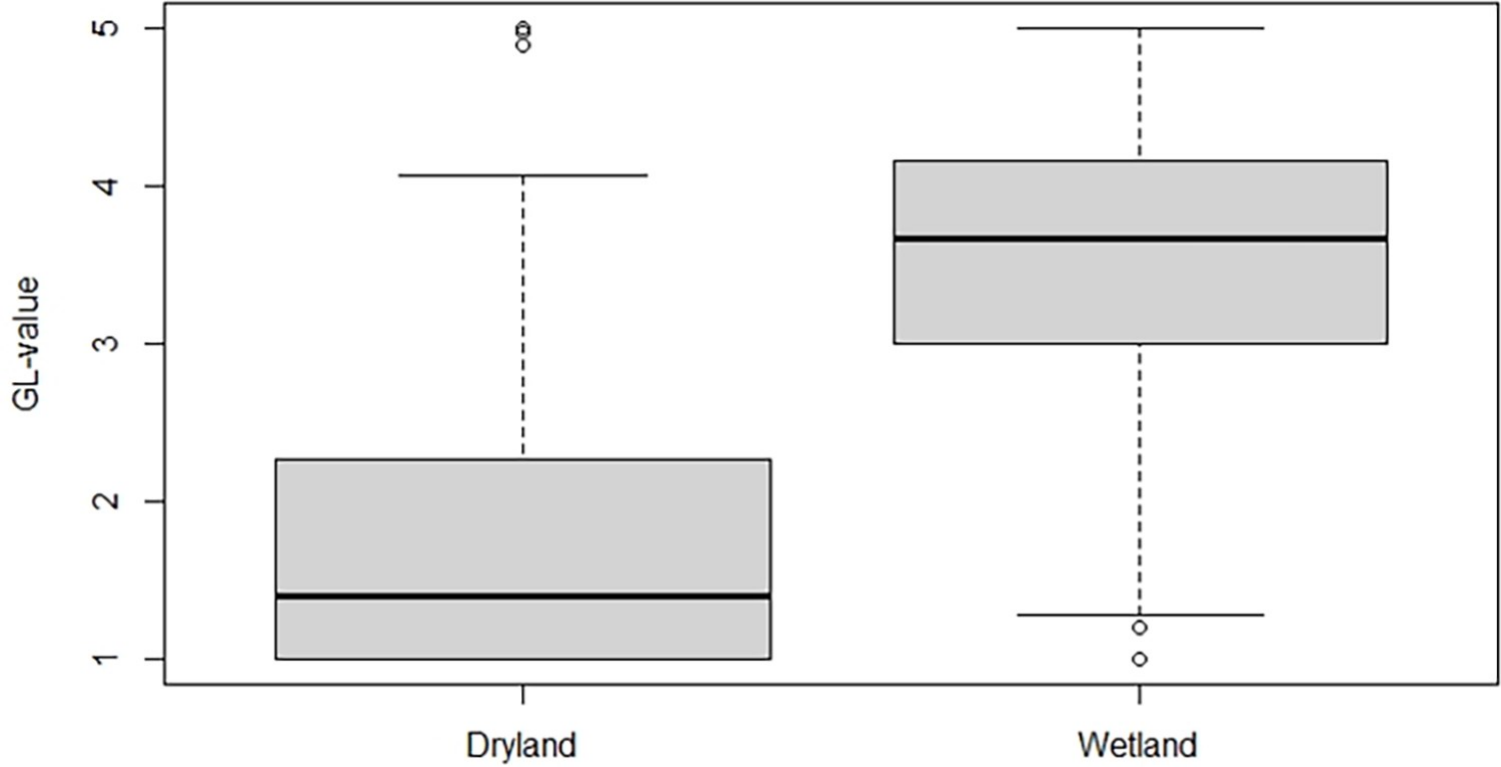
Comparison of land condition values (GL-values) for dryland sample areas and wetland sample areas in Iceland represented by boxplots.

Average land condition declined with increased elevation (Fig 7A). At lowland areas, most sites had land conditions values above 3, while above 700 m.a.s.l. all points had land conditions values under 3. The regression line for wetlands (Fig 7B) is characterized by large number of dryland sample areas (SA’s) on the left (low Wetland index) that have full range of GL-values ranging from barren surfaces (GL = 1) to lush systems (GL = 5). Comparing SA’s with some presence of wetlands (>5%) to those dry areas (<5% wetland) resulted in significant difference (t-test; t = -17.99, df = 395, p < 0.001; see Fig 8). It shows that the average GL-value is 3.5, which is considered good condition. The average value for all dryland SA’s is 1.8. Land condition declined with increased occurrence of dust deposition and steeper scree slopes (Fig 7). There were, however, large variation of conditions on flat surfaces and surfaces with little dust deposits.

### Results separated by regions

Models based on data from the South & West region and the North region explained more of the observed variability in land conditions than a model based on the whole country (Table 3).

The adjusted overall regression coefficient ranged from 0.636 for the peninsulas to 0.699 for the South and West. Elevation and the presence of wetlands (drainage) were generally the most influential variables, with lower land condition values at lower elevations and higher values at sites with higher percentage of wetlands, as was the case for the overall regression. Amount of dust, negatively affected land condition in all regions except in the peninsulas, where it had positive effects. Scree slopes were a significant contributor to the variability only for the North and the South & West regions, yet with considerably lower relationship compared to elevation, dust, and wetlands (Table 3).

There was a marked difference between the regression lines for elevation with the North and East regions sitting considerably higher than the South & West region and the peninsulas. Therefore the breaking point between poor (1 and 2) and marginal–excellent land conditions (3–5) differed. In the East this limit was at > 700 m elevation, at about 650 m for the North 500 m in the South & West and 400 m in the northern Peninsulas.

## Discussion

The test of the resilience-based model indicated that current ecosystem condition in Iceland is highly affected by elevation above sea level, proximity to the active volcanic zones, drainage (presence of wetlands) and slope characteristics. With the model explaining around 65% of the variability, it clearly explains the main factors influencing the current state of Icelandic ecosystems after experiencing continuous land pressures since the settlement around the year 900 AD, augmented by cooler climate during the Little Ice-Age and periodic volcanic eruptions.

### The elevation factor

**Fig 9:**
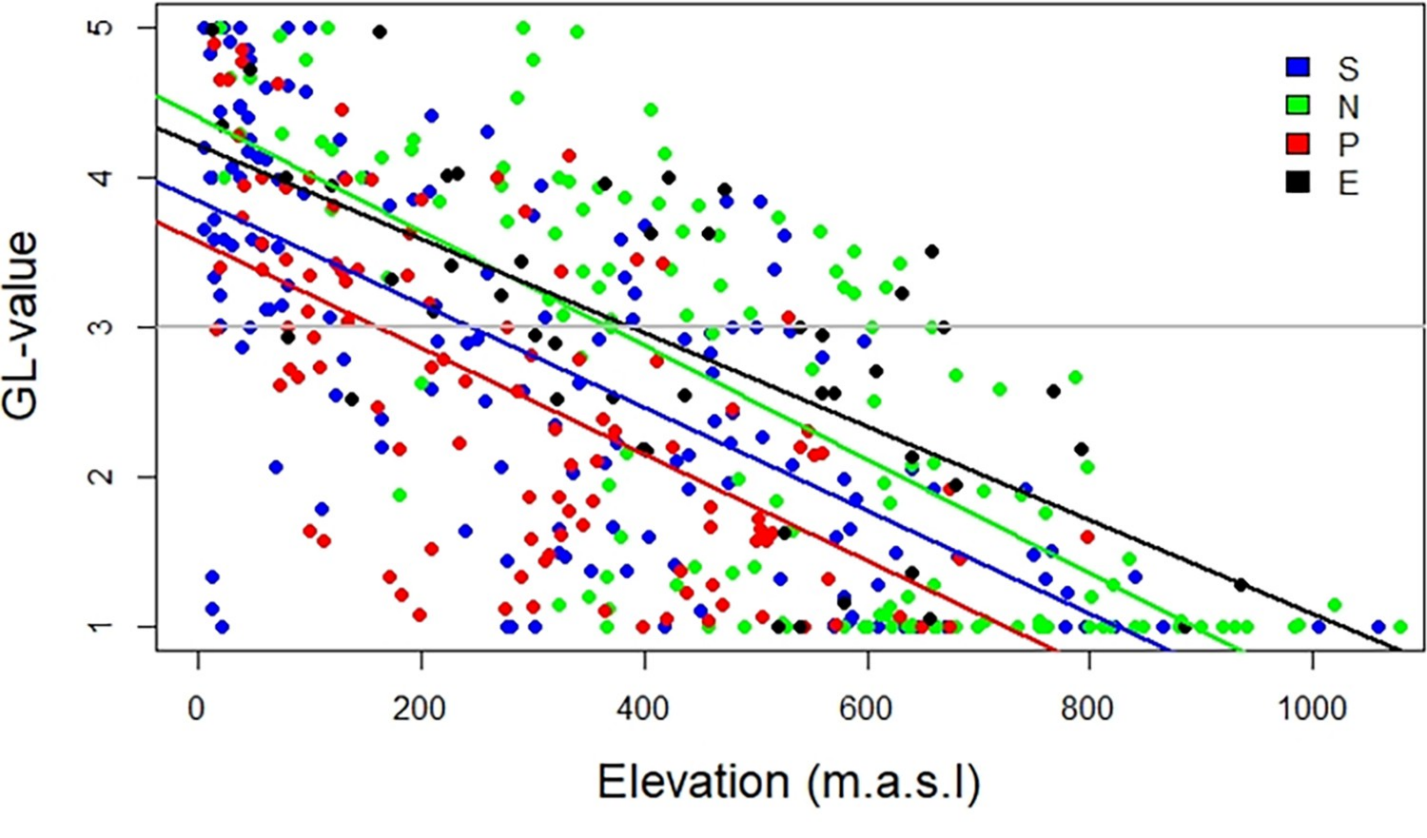
Relationship between GL-values (representing land conditions) and elevation in Iceland, separated by regions. The GL-values for the Westfords Peninsula and the northern peninsulas (P) are more sensitive to elevation (line sitting lower) than the North and East regions (N and E respectively), with the South & West (S) being intermediate. The difference is more than 200 m in elevation between the regression lines where it intercepts with the GL-value of 3, and even greater for lower GL-values. The vertical lines indicate elevations limits where sample areas (SA’s) with GL-values >3 (marginal to excellent condition).

Land condition in Iceland today, as represented by average GL-land condition values, are highly dependent on elevation (Fig 9). There is a clear cut-off at 700 m elevation in our data with all SA’s above this elevation with a lower GL-value than 3. Marginal or better GL-land condition values (3 or higher) are rarely present above 600 m elevation. This indicates that in general land condition is poor all over the country at higher elevations. This reflects heightened climatic constraints with increased elevation, which affect the resilience of ecosystems at higher elevations towards land use pressures exerted on the ecosystems after the settlement. The results provide a model-based underpinning for numerous studies that have emphasized the role of temperature and elevation in reducing plant growth in Iceland [58–60]. High elevation also makes systems more vulnerable to volcanic ash fall events and sand advancement over vegetation, because of lower vegetation height and other factors [44]. It should be noted that some areas at high elevations have limited vegetation cover without having been exposed to land use pressures over the past few centuries [7]. However, the extent of land use of these areas during the first centuries after the settlement is generally not known.

The relationship between elevation and land condition differed among regions. In the North and East, good land condition values can be found up to 600–700 m elevation while in the South & West and at the peninsulas this line is at 400–500 m elevation. This may be in part be a result of more continental climate in the North and East with higher summer temperature inlands, compared to the South & West [33]. These results are in line with findings by Traustason et al. [61] who studied the upper boundaries of vegetation outside the volcanic zones and found them as low as 300 m at the northernmost part of the Westfjord peninsula and at 450 m for the Tröllaskagi (Central-North Peninsula). Mean temperatures drop with proximity to the cold waters of the oceans north of Iceland, which explains relatively low GL-values for at low elevations of the peninsulas. There is also a large number of SA’s with GL-value of 1 (poorest condition) in the North, and in the South & West to some extent, most often reflecting barren areas, some of which are sandy deserts within or near the active volcanic zone, which bring the regression line somewhat down.

### Wetlands factor

The presence of wetlands, which are generally highly stable ecosystems, explains a substantial proportion of the variability of GL-values. The average GL-value for wetlands is near 4, which is considered good condition, while for all drylands it is 1.8 –which is poor condition–reaffirming the poor state of Icelandic ecosystems in general, especially when wetlands are excluded. It is noteworthy that wetlands still remain in the highlands, often surrounded by barren dryland positions, reiterating the high resilience of such systems. It should, however, be noted that some wetlands in Iceland have been totally desertified by various degradation processes, despite inherently higher resilience than other land ecosystems. These include sand burial associated with catastrophic flooding in glacial rivers during volcanic eruptions [7]. Such sandy surfaces are currently not classified as wetlands–they are sandy deserts. Yet, upon restoration, they gradually develop into wetlands again, as witnessed in many areas in South Iceland [7]. In these cases, our model does not account for wetlands lost to severe degradation processes. However, the box-plot in Fig 8 calls out the dramatic difference in land condition between drylands and wetlands.

### Slope factors

Factoring in the mean angle of slopes did not improve the overall prediction of the model, with a non-significant relationship. This is most likely due to the fact that the slope, as determined here for the SA’s, is based on height difference on the landscape which does not take barriers that shorten the length of slopes into consideration. Such barriers that split up long slopes into relatively short segments are common within the Tertiary basalt stacks. Many of the SA’s defined as having steep slopes in the present study have such barriers reducing the continuity of the slopes. Another possible reason for poor performance of the slope angle factor is that it does not weigh slope forms–i.e. if slopes are convex or concave in shapes. Both these factors, shape and the continuity of the slopes, are at the core of common water erosion models such as the USLE [49]. The presence of scree slopes did, however, yield a significant relationship (Fig 7D, Table 3). These slopes tend to be long and continuous as explained in the method section. It should be noted that present method only evaluates where scree is currently present, but not slopes that would become denuded scree slopes with continued land degradation processes. The model does, however, clearly indicate the importance of such slopes for understanding land degradation in Iceland.

### Dust deposition

We used dust sedimentation rates on the scale 1 to 7, which also include thickening by frequent ashfall events, to reflect proximity to periodic volcanic ash-fall events and active sandy dust sources. An interesting expression of this concept is that reduced land use pressure following the Plague in 1402 resulted in slower dust sedimentation rates, reflecting more ecosystem stability in general [27]. In our study, we find a slightly higher, yet significant probability of poorer land condition (lower GL-values) with higher sedimentation rates (Fig 7). Higher sedimentation rates create thicker and more coarse-grained soils, which are more susceptible to severe erosion process when the ground cover is compromised by land use. Many of the interior areas within the volcanic region are barren deserts and fall within the highest dust category.

### Improvement of the model prediction

Our model explained around 65% of the variability found in the GL land condition values. The GL land condition values were sometimes considerably higher or lower than was found by investigating the SA’s on satellite images, which explains some of the variability not accounted by the data. Part of the deviation results from occasional errors observed in the habitat mapping which is one of the databases used for arriving at the GL-values.

The erosion data used as one of the components for creating the GL database is also subjected to errors for various reasons. Mapping of erosion was mainly undertaken between 1992 and 1996 and it is known that land condition has changed in many areas since that time. Deviations include both improvements and further degradation–i.e., errors can go both ways (based on recent observations by the authors). We conclude that the predictability of the model will most likely improve with further development of the input data of the model, including the GroLind database and erosion data and scales, which is currently being undertaken with extensive ground-checking and subsequent modifications under the GroLind project of the Icelandic Soil Conservation Service [11].

Volcanic activity has created extensive lava surfaces during Holocene, which range in age from > 10 000 years to quite recent. Only small proportion of the randomly placed SA’s landed on recent lavas such as those formed after the settlement. However, accounting for the age of the lavas could possibly improve the outcome of the model. Age of the lavas is often unknown except for the most recent lavas and map data for the lavas were not yet available in an accessible form to meet the requirements for use in this model. It should be noted that there is an interaction between rate of dust deposition and age of lavas, as dust buries lavas and facilitates ecosystem development if vegetation cover is relatively stable. Thus, complex interaction between age, lava surface type, deposition rates and sand advancement are expected. This could be a factor to investigate further in future attempts to improve the model.

### Implication for management

The results have implications for land use decisions, where the resilience of the ecosystems need to be considered in addition to present day vegetation cover. The results suggest that grazing of domestic animals, which is on top of natural herbivory by geese, swans, and ptarmigans [62], should be limited at higher elevations. From the data we suggest that the limits or restrictions should be set at relatively low elevations (<300 m) at the northernmost peninsulas and possibly 400–600 m elevation in other areas. Icelandic nature laws clearly state that nature should have the benefit of the doubt in land use decisions [63], and these findings can aid in suggesting elevation limits to grazing by domestic animals. Long continuous slopes have often become totally devoid of soil cover, exposing rocky scree surfaces. This suggests that areas subjected to the formation scree slopes should be managed carefully or protected from grazing. Wetlands have always been important for grazing practices throughout the ages and are likely to continue to be used as such without much damage to these robust systems. Yet, many of the lowland wetlands have been drained for hay-making and to improve grazing conditions–which does, however, cause CO_2_ release from the systems–which is not the focus of the present paper. Proximity to volcanoes clearly affects both resilience of ecosystems to disturbance and increases the number of disturbances caused by volcanic eruptions and wind erosion. It is advisable that a general strategy for agriculture in Iceland would aim to reduce the number of free-ranging livestock within these areas.

## Conclusions

Iceland is among the most affected countries from land degradation and collapse of ecosystems [6, 7, 9, 10]. However, present ecosystem condition varies from rating excellent with robust functional ecosystems to fully collapsed barren desert areas. Here we present a resilience-based condition model (RBC), a novel framework that helps explaining the variability in land condition in present day Iceland after about 1100 years of land use. In the future the accuracy of the model could been improved further with new and improved data, and further development of land databases in Iceland. The main factors of this framework RBC-model are elevation, the presence of wetlands, slope characteristics, and dust sedimentation rates. The model provides a tool for drawing conclusions from research on the state of the land and consequences of land use for over a Millenia, based on these factors. The model can be useful for explaining results for different severity of disturbance caused by land use over the past 1100 years, occurring and different timescales, with the least resilient systems being first to be affected. The least resilient systems are those of high elevation and made of dry coarse thick soils, especially on steep continuous slopes, while resilient systems, such as wetlands, still remain relatively intact in comparison.

## Supporting information

S1 Table Overview of indications for pronounced ecosystems changes following the settlement of Iceland. (PDF)

S1 Data Data used in this paper. (XLSX)
