# Supplementary material for: A framework model for current land condition in Iceland
Source: PLoS One. 2023 Jul 6;18(7):e0287764. doi: 10.1371/journal.pone.0287764 (PMC10325058; doi:10.1371/journal.pone.0287764)
Supplement: S1 Table — (PDF) [file pone.0287764.s001.pdf]

Supporting information

S1 Table. Overview of indications for pronounced ecosystems changes following the settlement of Iceland.

Type of source	Notes (numbers refer to references listed in table caption)
Old written documents	Sagas, annals, legal and other written documents[1, 2,3]
Vegetation and soil remnants	Old soils buried under sand, vegetation islands within largely barren areas etc [3]
Pollen research (soils and sediments)	Shows dramatic vegetation changes at the time of settlement[4,5,6]
Remnants of charcoal in current desert areas.	Birch was cut and placed in pits and burned to make charcoal[7]
Soil thickening rates (rates of dust deposition)	4-10 times faster after the settlement than before due to wind erosion of soils and gradually larger desert area [3,8]
Place names	Some suggest forests and other rich systems in present day severely degraded areas[1]
Soil and lake sediments	Indicate erosion rates and past environmental conditions[9,10,11,12]
Archeology	Multiple evidence of past riches and farms in presently severely degraded areas[5,13]
Areas naturally protected from grazing	Rich ecosystems on islands in large rivers and at inaccessible rocky terrain [4,14]
Vegetation succession following exclusion from grazing	If conditions allow (e.g., seed availability and surface stability), vegetation, including birch, can spread over large areas. Large scale improvement often follows[2, 15]
Modelling of former birch distribution based on climate and other factors	Indicates wide spread of birch forests and shrubs before the settlement[16]
Multiple source approaches	Integrated approach. Combining some or all of the above[12,17]

References

1. Arnalds A. Ecosystem disturbance in Iceland. Arct Antarct Alp Res. 1987;19: 508-13. https://doi.org/10.2307/1551417
2. Sigurmundsson FS, Gísladóttir G, Óskarsson H. Decline of birch woodland cover in Þjórsárdalur Iceland from 1587 to 1938. Hum Ecol. 2014; 42: 577-90. https://doi.org/10.1007/s10745-014-9670-8
3. Arnalds Ó. The soils of Iceland. The Netherlands: Springer Netherlands; 2015.
4. Hallsdóttir M, Caseldine CJ. The Holocene vegetation history of Iceland, state-of-the-art and future research. In: Caseldine C, Russel A, Harðardóttir J, Kudsen O, editors. Iceland – Modern Processes and Past Environments. Developments in Quaternary Science 5. Amsterdam: Elsevier. pp. 319-334. https://doi.org/10.1016/S1571-0866(05)80016-8
5. Erlendsson E, Edwards KJ, Buckland PC. Vegetational response to human colonisation of the coastal and volcanic environments of Ketilsstaðir, southern Iceland. Quaternary Research. 2009;72: 174-87. https://doi.org/10.1016/j.yqres.2009.05.005
6. Einarsson Th. Vitnisburður frjógreiningar um gróður, veðurfar og landnám á Íslandi. Saga. 1962, 442-469. Icelandic
7. Arnalds, ÓO. (2020). Land condition and the collaps of Icelandic ecosystems [Astand lands og hrun islenskra vistkerfa]. In Icelandic. Hvanneyri: Agricultural University of Iceland; 2020. Agricultural University of Iceland Reprort no. 130.. Hvanneyri, Reykjavik. Icelandic.
8. Thorarinsson, S. (1961). Uppblastur a Islandi i ljosi öskulagarannsokna. Arsrit Skogræktarfelags Islands 1960-1961, 17-54.Thórarinsson S. Uppblástur á Íslandi í ljósi öskulagarannsokna. Ársrit Skógræktarfélags Íslands 1960-1961, 1961; 17-54. Icelandic.
9. Geirsdóttir A, Miller GH, Thórðarson T, Ólafsdóttir KB. A 2000 year record of climate variations reconstructed from Haukadalsvatn, West Iceland. J Paleolimnol. 2009;41: 95-115. https://doi.org/10.1007/s10933-008-9253-z
10. Guðbergsson, G. (1996). I norðlenskri vist. Um gróður, jarðveg, búskaparlög og sögu. Icel Agric Sci. . Icelandic Agricultural Sciences1996; 10:, 31–89. Icelandic

11. Ólafsdóttir R, Guðmundsson HJ. Holocene land degradation and climatic change in northeastern Iceland. Holocene. 2002;12: 159-67. https://doi.org/10.1191/0959683602hl531

12. McGovern TH, Vésteinsson O, Friđriksson A, Church M, Lawson I, Simpson IA, Einarsson A, Dugmore A, Cook G, Perdikaris S, Edwards KJ. Landscapes of settlement in northern Iceland: Historical ecology of human impact and climate fluctuation on the millennial scale. Am Anthropol. 2007;109: 27-51. https://doi.org/10.1525/aa.2007.109.1.27

13. Lawson IT, Gathorne-Hardy FJ, Church MJ, Newton AJ, Edwards KJ, Dugmore AJ, Einarsson A. Environmental impacts of the Norse settlement: palaeoenvironmental data from Myvatnssveit, northern Iceland. Boreas. 2007 ;36: 1-9. https://doi.org/10.1111/j.1502-3885.2007.tb01176.x

14. Jónsdóttir IS. Áhrif beitar á gróður Auðkúluheiðar. Náttúrufræðingurinn. 1985; 53: 19–40.

15. Marteinsdóttir B, Svavarsdóttir K, Thórhallsdóttir TE. Multiple mechanisms of early plant community assembly with stochasticity driving the process. Ecology. 2018;99: 91-102. https://doi.org/10.1016/j.rama.2021.04.006

16. Ólafsdóttir R, Schlyter P, Haraldsson HV. Simulating icelandic vegetation cover during the holocene implications for long-term land degradation. Geogr Ann Ser A. 2001;83: 203-15. https://doi.org/10.1111/j.0435-3676.2001.00155.x

17. Vickers K, Erlendsson E, Church MJ, Edwards KJ, Bending J. 1000 years of environmental change and human impact at Stóra-Mörk, southern Iceland: a multiproxy study of a dynamic and vulnerable landscape. The Holocene. 201;21: 979-95. https://doi.org/10.1177/0959683611400201
